# The Effects of Electron-Beam-Radiation-Induced Damage on Single-Crystal Silicon Devices with SiO_2_ Surface Passivation in a Nitrogen Atmosphere

**DOI:** 10.3390/ma19101964

**Published:** 2026-05-10

**Authors:** Yuqing Yang, Yisong Lei, Xinxi Li, Wenzeng Bing, Hongbo Li, Yongjun Xiang, Shuming Peng

**Affiliations:** 1 Institute of Nuclear Physics and Chemistry, China Academy of Engineering Physics, Mianyang 621900, China; yangyuqing20@gscaep.ac.cn (Y.Y.); leiyisongm3@163.com (Y.L.); hkllxx@sina.com (X.L.); bwz@caep.cn (W.B.); 1234lihongbo@163.com (H.L.); 2 The 44th Research Institute of China Electronics Technology Group Corporation, Chongqing 400060, China; 18523977510@163.com

**Keywords:** β-voltaic battery, damage effects, electron beam radiation, SiO_2_/Si interface, nitrogen atmosphere, neutron reflectometry, X-ray photoelectron spectroscopy, secondary ion mass spectrometry

## Abstract

In energy conversion semiconductor devices, radiation damage is directly related to the long-term stability of β-voltaic batteries. In this study, single-crystalline silicon P^+^NN^+^ devices and P^+^-silicon materials with SiO_2_ surface passivation were irradiated using a ~70 keV accelerator electron beam in a nitrogen atmosphere for 2 min, 10 min, 1 h, 6 h, and 12 h. The tritium-voltaic output decreased rapidly within the first 2 min of electron beam irradiation and then decayed slowly. After 1 h of irradiation, both the output short-circuit current (Isc) and open-circuit voltage (Voc) remained stable. The effects of the damage were analyzed using typical samples irradiated for 1 h. Neutron reflectometry (NR) was employed as the primary characterization method, while X-ray photoelectron spectroscopy (XPS)—combined with Ar^+^ etching—and secondary ion mass spectrometry (SIMS) were used to verify radiation-induced structural changes at the SiO_2_ surface and SiO_2_/Si interface. It was found that nitrogen atoms from the atmosphere penetrated the SiO_2_ layer to a depth of approximately 5–10 nm, forming a non-stoichiometric SiON structure, without further diffusion into deeper layers. Irradiation significantly increased the thickness of the SiO_2_/Si interface transition layer to about 14–18.5 nm, and the SiO_2_ structure within this layer became relatively loose. It can be inferred that tritium-voltaic batteries using SiO_2_-surface-passivated single-crystalline silicon P^+^NN^+^ devices as energy-conversion units and packaged in a nitrogen atmosphere can stably provide power for 10 years, with an Isc reduction of no more than 12% and a Voc reduction of no more than 6%, excluding the spontaneous decay of tritium.

## 1. Introduction

Radio-voltaic isotope batteries are long-life, low-power sources suitable for micro-systems where battery replacement is difficult [1,2,3]. This field has been a research hotspot over the past 30 years; however, the development of long-life radio-voltaic batteries is hampered by numerous challenges [4,5]. Such batteries typically employ low-energy beta emitters with relatively low energy for convenient radiation shielding, such as tritium and nickel-63 [6,7,8]. Wide-bandgap semiconductors used as energy-conversion devices for beta-voltaic batteries offer advantages such as high-temperature resistance and radiation tolerance [9,10]. Nevertheless, single-crystalline silicon devices remain the most important components in integrated circuits (ICs) and micro-electro-mechanical systems (MEMSs). For radio-voltaic batteries that can be monolithically integrated with single-crystalline silicon MEMS devices, single-crystalline silicon devices represent the ideal energy-conversion units.

However, single-crystalline silicon devices are susceptible to radiation damage [11]. To predict the long-term output stability of β-voltaic batteries based on tritium or nickel-63 and single-crystalline silicon devices, accelerator electron beams with comparable energy are commonly used to investigate the accelerated radiation effects [12]. Although the threshold displacement energy of silicon is approximately 250 keV [13], which is much higher than the maximum beta electron energy of tritium or nickel-63, damage can be inflicted at the interfaces between passivation layers and silicon substrates in PN-junction devices for tritium-voltaic batteries [14]. Meanwhile, the radiation atmosphere significantly influences damage behavior. Single-crystalline silicon devices are particularly prone to failure when irradiated in air due to reactions with oxygen and water vapor [15]. It is well known that highly reliable single-crystalline silicon devices are usually packaged in dry nitrogen to isolate moisture and oxygen. However, few studies have been conducted on single-crystalline silicon devices irradiated in a nitrogen atmosphere.

In this work, single-crystalline silicon devices and materials with SiO_2_ surface passivation were irradiated using a ~70 keV accelerator electron beam in a nitrogen atmosphere. The effects of electron-irradiation-induced damage on the devices and materials were analyzed, including tritium-voltaic performance before and after irradiation using a tritium experimental source, and the surface/interface chemical microstructures before and after irradiation using neutron reflectometry (NR). NR was used as the primary technique because it avoids additional disturbance of the radiation-sensitive regions of silicon devices or materials during measurement. Furthermore, to validate the structural changes on surfaces and at interfaces identified by NR, X-ray photoelectron spectroscopy (XPS) with Ar^+^ etching and secondary ion mass spectrometry (SIMS) were applied to the irradiated samples.

## 2. Materials and Methods

**Figure 1:**
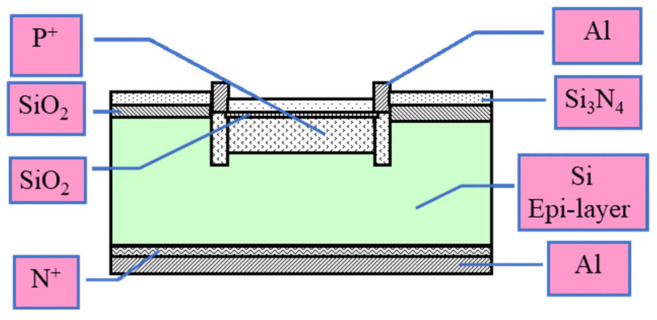
Schematic of the structure of the SiO_2_/P^+^NN^+^ device.

Single-crystal silicon epitaxial wafers were provided by Zhejiang Jinruihong Technology Co., Ltd. (Ningbo, China). SiO_2_-surface-passivated (40 ± 2 nm) single-crystal silicon P^+^NN^+^ devices (280 μm thick) with an active area of Φ = 1 cm and SiO_2_-surface-passivated (40 ± 2 nm) single-crystalline silicon P^+^-silicon materials (<100>, 5 cm × 5 cm × 900 μm, 0.01 Ω cm) were fabricated at the 44th Research Institute of China Electronics Technology Group Corporation (Chongqing, China). The SiO_2_ passivation layer was prepared using a gate oxidation process with dry oxygen oxidation. A schematic of the structure of the device is shown in Figure 1.

Silicon devices and materials were irradiated using a ~70 keV accelerator electron beam under flowing nitrogen for 1 h. The characteristics of the accelerator electron beam were identical to those reported in previous work [14].

**Figure 2:**
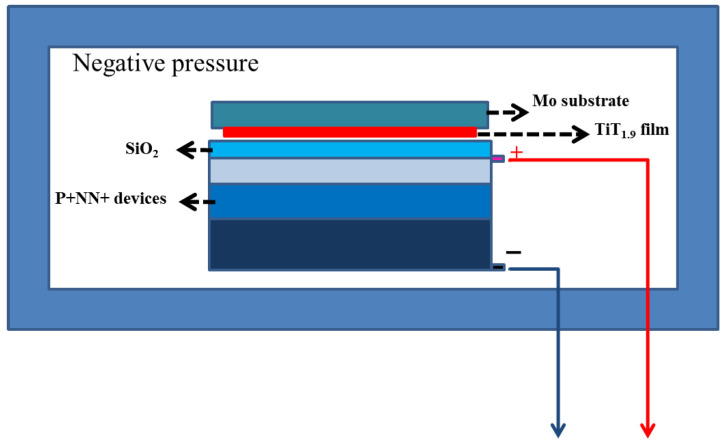
Schematic of the tritium-voltaic output performance test.

The tritium-voltaic response of the silicon P^+^NN^+^ devices was measured using the same experimental setup as employed in previous studies [16]. The surface of the TiH_1.9_ source faced the active area of the single-crystal silicon device, with a fixed relative position. The assembly was placed in a negative pressure tank maintained at −92.8 kPa, and the positive and negative electrodes of the silicon device were left outside the tank for testing. A schematic of the tritium-voltaic performance test is shown in Figure 2. All experiments were performed using duplicate parallel samples.

Neutron reflectometry (NR) is a non-destructive technique that utilizes the interactions between neutron spins, a sample, and external magnetic fields [17,18]. The depth-resolved structural properties of the silicon P^+^-silicon materials were characterized using a polarized neutron reflectometer in time-of-flight mode in Mianyang, China (Diting (TPNR)).

X-ray photoelectron spectroscopy (XPS) (Thermo Fisher Scientific, Hillsboro, OR, USA) is widely used to characterize the surfaces and interfaces of silicon and its compounds [19,20]. XPS measurements were performed using an ESCALAB250Xi spectrometer with a monochromatized Al Kα X-ray source (1486.6 eV) (Thermo Fisher Scientific, Hillsboro, OR, USA). XPS spectra were recorded with an analyzer pass energy of 30 eV, and the overall energy resolution for core-level photoemission spectra was 0.45 eV. The X-ray spot size was ~500 μm, and an electron take-off angle of 90° was used. The hemispherical analyzer was operated at 20 eV pass energy in standard magnetic lens mode. To mitigate differential electrostatic charging, samples were measured under floating conditions, and an in-lens flood gun was operated at 4 eV. The instrument binding energy scale was calibrated using Au 4f_7_/_2_ = 83.96 eV, Ag 3d_5/2_ = 368.21 eV, and Cu 2p_3/2_ = 932.62 eV. Ar^+^ etching was performed at 1 kV with a flux of 1 μA. During data analysis, the maximum binding energy of the C 1s peak at each etching step was set to 284.8 eV to correct the binding energies of O 1s, N 1s, and Si 2p at the same depth.

Secondary ion mass spectrometry (SIMS) has long been an important technique for depth-profiling semiconductor devices [21,22]. SIMS measurements were carried out using a TOF SIMS 5 instrument (ION-TOF GmbH, Münster, Germany). Cs^+^ ions at 1 kV with a flux of 65 nA were used for etching, and Ga^+^ ions at 25 kV with a flux of 25 nA were used for analysis. The etching area was 400 μm × 400 μm, and the analysis area was 100 μm × 100 μm.

## 3. Results

### 3.1. Tritium-Voltaic Performance of Devices Before and After Irradiation

**Figure 3:**
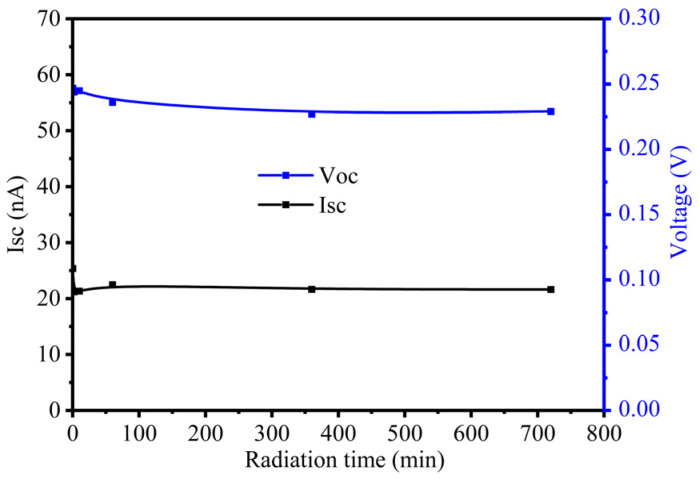
Variations in tritium-voltaic output parameters Isc and Voc of SiO_2_-passivated P^+^NN^+^ devices with irradiation time in a nitrogen atmosphere.

SiO_2_-passivated P^+^NN^+^ devices were irradiated in a nitrogen atmosphere for 2 min, 10 min, 1 h, 6 h, and 12 h. Tritium-voltaic short-circuit current (Isc) and open-circuit voltage (Voc) were measured after irradiation. The variations in Isc and Voc with irradiation time are shown in Figure 3. As shown in Figure 3, the tritium-voltaic output decreases rapidly within the first 2 min of electron beam irradiation and then decays slowly. After 1 h of irradiation, both Isc and Voc stabilize, and all subsequent analyses were performed using typical samples irradiated for 1 h.

**Figure 4:**
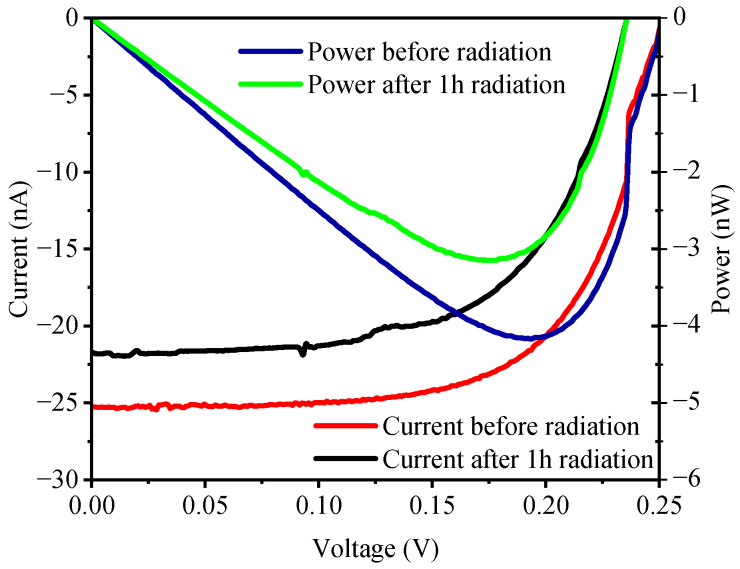
Tritium-voltaic output I-V curves of SiO_2_-surface-passivated single-crystal silicon P^+^NN^+^ devices before and after 1 h of electron irradiation in a nitrogen atmosphere.

A comparison of tritium-voltaic output I-V curves is shown in Figure 4. As can be observed, the tritium-voltaic Isc, Voc, maximum output power (Pmax), and fill factor (FF) of the SiO_2_-passivated P^+^NN^+^ device all degrade after 1 h of irradiation under flowing nitrogen. The Isc and Voc drop by approximately 12% and 6%, respectively. The duplicate parallel samples show excellent consistency: the relative deviations of Isc and Voc were 0.4% and 0.8% before irradiation and 3.8% and <0.1% after irradiation, respectively. The Pmax values before and after irradiation were 4.18 nW and 3.15 nW, respectively, and the FF values were 0.656 and 0.616, respectively.

### 3.2. NR Analysis of SiO_2_-Surface-Passivated P^+^-Silicon Materials Before and After Irradiation

**Figure 5:**
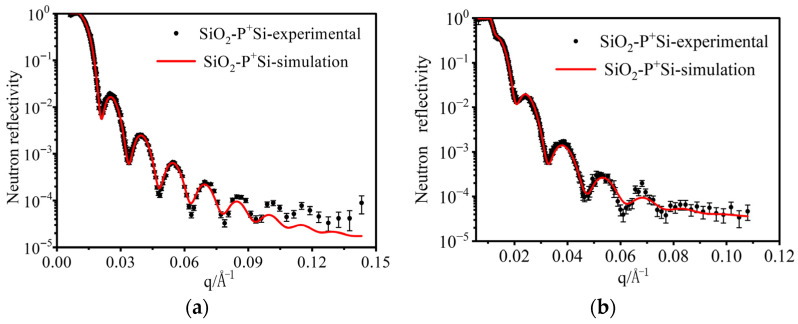
NR spectra and fitted curves for samples (**a**) before and (**b**) after irradiation.

**Figure 6:**
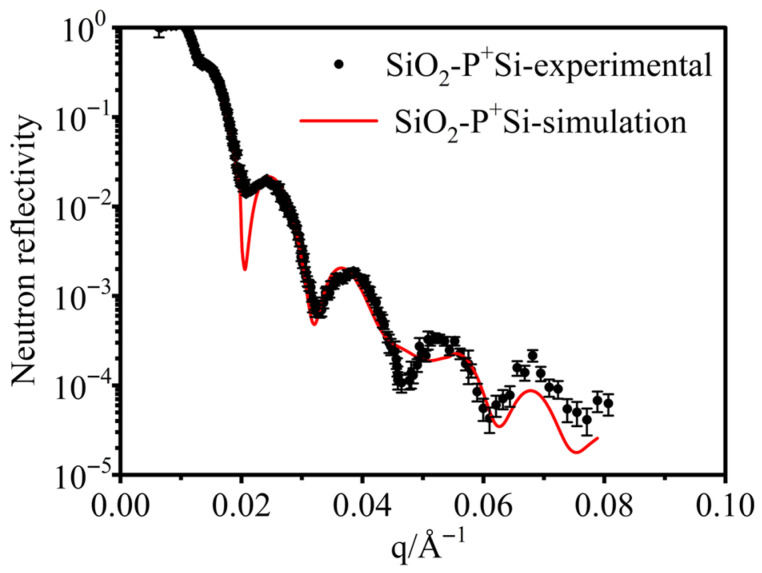
NR spectra and fitted curves of samples for irradiated samples with the surface layer modeled as pure SiO_2_.

Neutron reflectometry was performed on SiO_2_-surface-passivated P^+^-silicon materials before and after irradiation under the same conditions (Figure 5). The fitted structural parameters of the surface passivation layers are listed in Table 1 and Table 2. During NR analysis, if the surface layer was assumed to be pure SiO_2_, the simulated curve deviated significantly from the experimental curve, as shown in Figure 6. Note that the term “roughness” in Table 1 and Table 2 is better interpreted as a structurally disordered transition layer with an ill-defined composition.

### 3.3. Ar^+^-Etched XPS Analysis of SiO_2_-Surface-Passivated P^+^–Silicon Materials Before and After Irradiation

**Figure 7:**
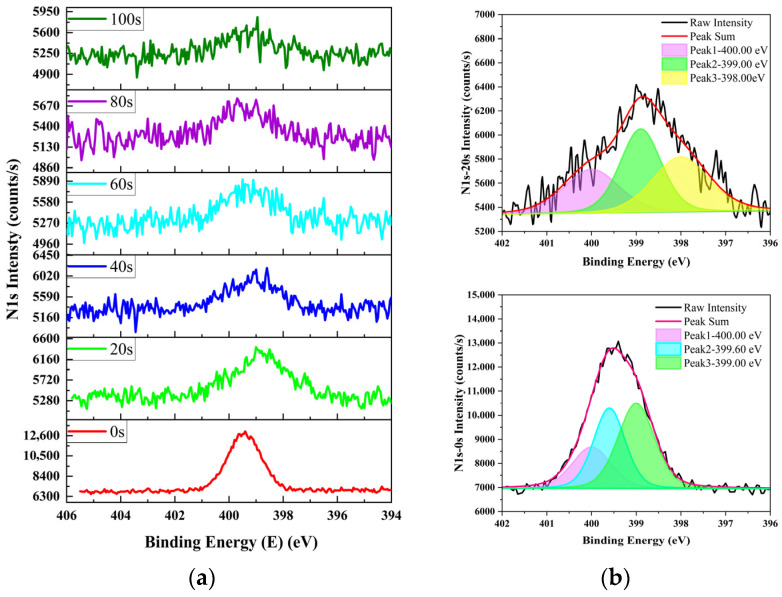
N 1s XPS spectra of Ar^+^-etched SiO_2_-surface-passivated P^+^–silicon after irradiation: (**a**) evolution over etching time, and (**b**) peak fitting at 0 s and 20 s of etching.

**Figure 8:**
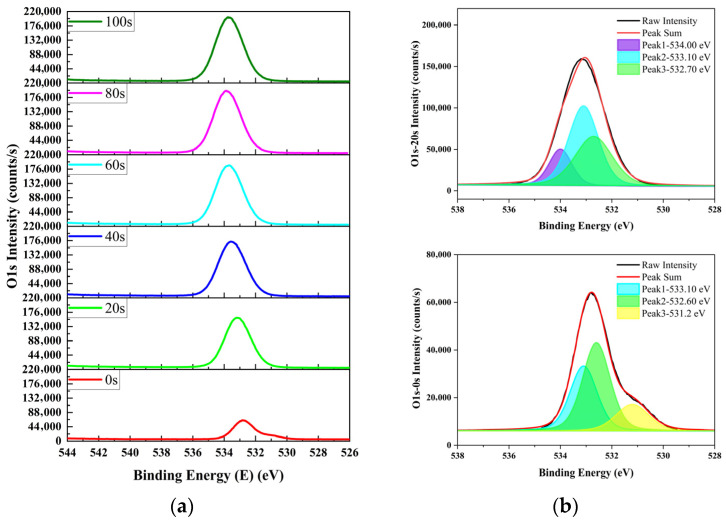
O 1s XPS spectra of Ar^+^-etched SiO_2_-surface-passivated P^+^–silicon after irradiation: (**a**) evolution over etching time and (**b**) peak fitting at 0 s and 20 s of etching.

**Figure 9:**
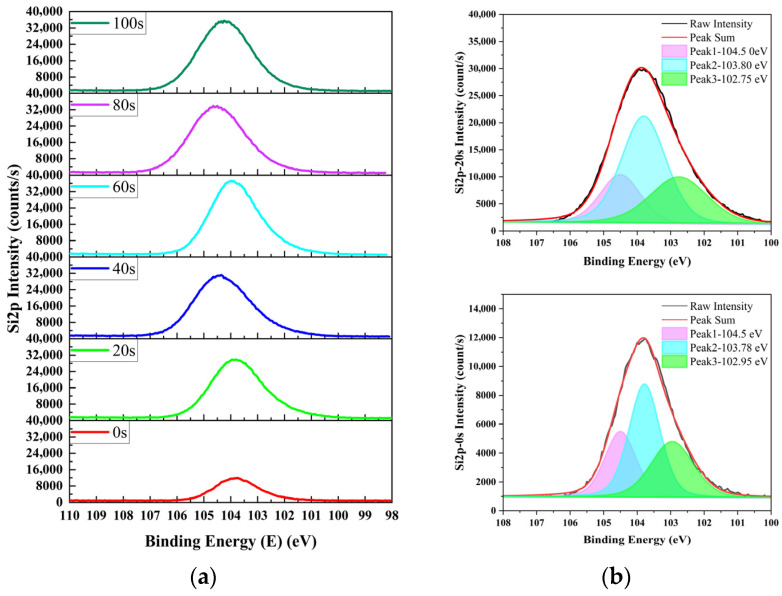
Si 2p XPS spectra of Ar^+^-etched SiO_2_-surface-passivated P^+^–silicon after irradiation: (**a**) evolution over etching time and (**b**) peak fitting at 0 s and 20 s of etching.

Parallel samples from same batch used for NR analysis were characterized via Ar^+^-etched XPS. The N 1s, O 1s, and Si 2p spectra of the irradiated samples are shown in Figure 7, Figure 8 and Figure 9, respectively.

### 3.4. SIMS Analysis of SiO_2_-Surface-Passivated P^+^–Silicon Materials Before and After Irradiation

**Figure 10:**
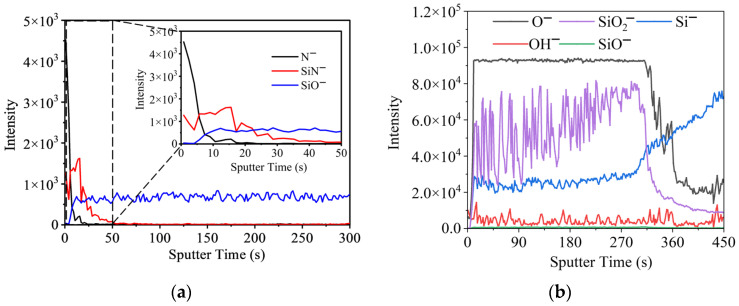
SIMS depth profiles of irradiated SiO_2_-surface-passivated P^+^–silicon: (**a**) distributions of N^−^, SiN^−^, and SiO^−^, and (**b**) distributions of O^−^, OH^−^, Si^−^, SiO^−^, and SiO_2_^−^.

SIMS analysis was performed on the same samples characterized via Ar^+^-etched XPS. The depth distributions of surface and interface ions are shown in Figure 10.

## 4. Discussion

The penetration depth of ~70 keV electrons in silicon and SiO_2_ is approximately 20 μm, so accelerated electrons easily pass through the 40 ± 2 nm SiO_2_ passivation layer and penetrate silicon P^+^NN^+^ devices or materials.

As shown in Table 1 and Table 2, irradiation significantly increases the thickness of the SiO_2_/Si interface transition layer. The low scattering length density (SLD) of the SiO_2_-Si transition layer indicates that its atomic structure is loose and defective. In addition, a new thin surface layer with high SLD forms on the SiO_2_ surface. The bond energy of N≡N is 9.8 eV, while the electron energy used in this experiment was ~70 keV, which is sufficient to turn nitrogen molecules into reactive nitrogen species N*. This process is more readily induced by electron irradiation than by high-temperature heating [23]. Subsequently, some O-Si-O bonds at the SiO_2_ surface are broken and react with N* to form a non-stoichiometric Si-O-N structure. The incorporation of nitrogen atoms increases the SLD of this surface layer and significantly increases surface roughness.

As shown in Figure 7, Figure 8 and Figure 9, the surface O 1s and Si 2p intensities are markedly lower than those in the etched subsurface region, whereas the surface N 1s intensity is substantially higher. This observation confirms that nitrogen atoms were incorporated into the near-surface SiO_2_ layer. The binding energy of the detected N 1s is much lower than that of physisorbed nitrogen (402.8 eV) [24], indicating that nitrogen atoms were chemically bonded to Si and O in the SiO_2_ matrix rather than physically adsorbed.

As shown in Figure 7, for the sampled etched for 0 s, the N 1s peak intensities are in the following order: 399.0 eV > 399.6 eV > 400.0 eV. For the sample etched for 20 s, the order is 399.6 eV > 399.0 eV > 400.0 eV. These distinct peaks correspond to different SiON bonding configurations and chemical states [24,25], where 399.0 eV is assigned to a higher nitrogen bonding ratio and 400.0 eV to a lower ratio. Compared with the sample etched for 0 s, the N 1s peaks of the sampled etched for 20 s are obviously broadened, revealing more complex and diverse SiON-bonding environments in the subsurface region than at the surface.

As shown in Figure 8, the O 1s spectrum of the sample etched for 0 s shows an additional peak at 531.2 eV relative to the sample etched for 20 s. Based on references [26,27], this peak can be attributed to oxygen vacancies or interstitial oxygen induced by irradiation and extensive nitrogen incorporation. In the O 1s spectra, the peak at 533.1 eV corresponds to oxygen in stoichiometric SiO_2_, and the peak at 532.6 eV can be assigned to oxygen in non-stoichiometric SiON [24]. The intensity order for the sample etched for 0 s is 532.6 eV > 533.1 eV > 531.2 eV, confirming the formation of an abundance of non-stoichiometric SiON in the surface layer.

For the sample etched for 20 s, the O 1s peaks at 532.7 eV and 533.1 eV can be attributed to oxygen in non-stoichiometric SiON and stoichiometric SiO_2_, respectively. The absence of the 531.2 eV peak indicates that oxygen vacancies or interstitial oxygen are no longer present in the inner layer due to reduced nitrogen incorporation. The weak peak at 534.0 eV may be related to partially dangling SiO_x_ species (x < 2). The intensity order for the sample etched for 20 s is 533.1 eV > 532.7 eV > 534.0 eV.

As shown in Figure 9, the Si 2p spectra of the samples etched for 0 s and 20 s contain three characteristic components: 103.78–103.8 eV, 102.75–102.95 eV, and 104.5 eV. Among them, 103.78–103.8 eV corresponds to Si in stoichiometric SiO_2_, 102.75–102.95 eV corresponds to Si in non-stoichiometric SiON, and 104.5 eV corresponds to partially dangling SiO_x_ (x < 2). This result is consistent with the presence of oxygen vacancies or interstitial oxygen in the sample etched for 0 s, which generate oxygen-deficient defect states (i.e., SiOₓ, x < 2) in partial SiO_2_ regions.

Furthermore, the Si 2p peak intensity order for the sample etched for 0 s is 103.78 eV > 104.5 eV > 102.95 eV, whereas for the sample etched for 20 s it is 103.78 eV > 102.75 eV > 104.5 eV. All Si 2p peaks for the sample etched for 20 s exhibit larger full-width-at-half-maximum (FWHM) values than those for the sample etched for 0 s. This further confirms that the SiON bonding environment is more complex in the subsurface region than at the surface, consistent with the trend observed in N 1s spectra.

In addition, obvious shifts in the O 1s and Si 2p peaks toward higher binding energies were observed following an increase in etching depth. This phenomenon requires further investigation in future work.

As shown in Figure 10, a clear transition layer for O^−^ appears at an etching time of approximately 300~400 s, and a distinct transition layer for Si^−^ can be observed at approximately 284~446 s. Based on the SiO_2_ thickness of 38.5 nm resulting from the fabrication process parameters, the SIMS etching rate was estimated to be approximately 1 nm per 10 s. Thus, the thickness of the transition layer in the 100 μm × 100 μm SIMS analysis area is about 10~16.2 nm. This is consistent with the SiO_2_/Si interface roughness of 18.5 nm and the SiO_2_–Si transition layer thickness of 14.1 nm determined via NR.

Furthermore, based on the SIMS etching rate of ~1 nm per 10 s and the observation that the SiN^−^ signal dropped to the background level after 55 s of etching (Figure 10a), the thickness of the nitrogen-containing surface layer was conservatively estimated to be no more than 10 nm. This is also consistent with the NR results, which reveal a 10.6 nm thick Si–N–O surface layer with a roughness of 7.7 nm.

It should be noted that NR, XPS, and SIMS analyze significantly different areas. NR probes the entire 5 cm × 5 cm × 900 μm sample, XPS analyzes a circular area with a diameter of ~500 μm, and SIMS analyzes a 100 μm × 100 μm square area. Because the area in NR analysis is much larger than in XPS and SIMS, NR averages out thickness inhomogeneities across the sample. Therefore, it is reasonable to assume that the thicknesses of the Si-SiO_2_ transition zone and nitrogen-containing surface zone determined by NR are larger than those obtained by SIMS.

It should also be noted that, for thickness analysis of multilayer complex samples using etched XPS and SIMS without standard calibration samples, the etching depth calculated from etching time is inherently semi-quantitative; however, qualitative analysis is reliable.

When SiO_2_-passivated P^+^NN^+^ devices are irradiated by electron beams in a nitrogen atmosphere, two competing effects occur. On the one hand, the formation of SiON near the SiO_2_ surface produces a passivation effect similar to that of a Si_3_N_4_/SiO_2_ composite structure, forming a denser surface passivation film that reduces surface leakage current and slows the decay in tritium-voltaic voltage. On the other hand, irradiation induces a pronounced, relatively loose transition layer at the Si-SiO_2_ interface with high structural defect density, which enhances interface recombination and reduces the tritium-voltaic output current.

Despite these significant structural changes, the tritium-voltaic output degradations of 12% for Isc and 6% for Voc are acceptable, especially when compared with the standard that allows current attenuation of less than 15% for space solar cells.

The electron beam flux rate used in this study is 6540 times that of a tritium source, and the total electron fluence after 12 h of irradiation is equivalent to 10 years of continuous tritium source irradiation. As shown in Figure 3, the tritium-voltaic output after 1 h of irradiation is nearly identical to that after 12 h of irradiation. Meanwhile, electron beam irradiation caused more severe damage than tritium source irradiation. Therefore, it can be inferred that tritium-voltaic batteries using SiO_2_-surface-passivated single-crystal silicon P^+^NN^+^ devices as energy-conversion units and packaged in a nitrogen atmosphere can provide stable electrical power for 10 years, with an Isc reduction of no more than 12% and a Voc reduction of no more than 6%, excluding the natural decay of tritium.

## 5. Conclusions

When single-crystal silicon P^+^NN^+^ devices and P^+^-silicon materials with SiO_2_ surface passivation are irradiated by a ~70 keV accelerator electron beam in a nitrogen atmosphere, the tritium-voltaic output decreases rapidly within the first 2 min and then decays slowly. After 1 h of irradiation, both Isc and Voc stabilize. Damage effects were analyzed using typical samples irradiated for 1 h. NR was used as the primary characterization method, while XPS with Ar^+^ etching and SIMS were used to verify radiation-induced structural changes at the SiO_2_ surface and SiO_2_/Si interface. During irradiation, nitrogen atoms from the atmosphere are activated and incorporated into the SiO_2_ surface layer down to a depth of approximately 5–10 nm, forming a non-stoichiometric SiON structure. After 1 h of irradiation, the SiO_2_/Si interface transition layer thickens significantly to about 14–18.5 nm, and the SiO_2_ structure within this layer becomes relatively loose. The near-surface SiON layer may form a denser passivation film that reduces surface leakage current and mitigates voltage decay. The loose interfacial transition layer enhances carrier recombination and reduces the output current. These combined surface and interface structural changes collectively regulate the β-voltaic output performance. The overall degradation in β-voltaic output is acceptable for long-life device applications.

## Figures and Tables

**Table 1:** Multilayer structural parameters of pristine SiO_2_-surface-passivated P+-silicon obtained via NR fitting.

Material	Thickness/Å	SLD/10^−8^ Å^−2^	Roughness/Å
SiO_2_	412	3.78	5
SiO_2_-Si	12	1.39	1
Si	Infinite	2.30	54

**Table 2:** Multilayer structural parameters of irradiated SiO_2_-surface-passivated P^+^-silicon obtained via NR fitting.

Material	Thickness/Å	SLD/10^−8^ Å^−2^	Roughness/Å
Unknown layer	106	5.78	77
SiO_2_	270	3.31	3
SiO_2_-Si	141	0.29	185
Si	Infinite	2.45	24

## Data Availability

The original contributions presented in this study are included in the article. Further inquiries can be directed to the corresponding author.

## Abbreviations

The following abbreviations are used in this manuscript:

NR	Neutron reflectometry
XPS	X-ray photoelectron spectroscopy
SIMS	Secondary ion mass spectrometry
IC	Integrated circuit
MEMS	Micro-electro-mechanical system
Pmax	Maximum power
FF	Fill Factor
